# Androgen and Progesterone Receptors Are Targets for Bisphenol A (BPA), 4-Methyl-2,4-bis-(P-Hydroxyphenyl)Pent-1-Ene—A Potent Metabolite of BPA, and 4-Tert-Octylphenol: A Computational Insight

**DOI:** 10.1371/journal.pone.0138438

**Published:** 2015-09-17

**Authors:** Mohd Rehan, Ejaz Ahmad, Ishfaq A. Sheikh, Adel M. Abuzenadah, Ghazi A. Damanhouri, Osama S. Bajouh, Samera F. AlBasri, Mansour M. Assiri, Mohd A. Beg

**Affiliations:** 1 King Fahd Medical Research Center, King Abdulaziz University, Jeddah, Kingdom of Saudi Arabia; 2 KACST Technology Innovation Center in Personalized Medicine, King Abdulaziz University, Jeddah, Kingdom of Saudi Arabia; 3 Department of Obstetrics and Gynecology, Faculty of Medicine, King Abdulaziz University, Jeddah, Kingdom of Saudi Arabia; Hormel Institute, University of Minnesota, UNITED STATES

## Abstract

Exposure to toxic industrial chemicals that have capacity to disrupt the endocrine system, also known as endocrine disrupting chemicals (EDCs), has been increasingly associated with reproductive problems in human population. Bisphenol A (BPA; 4,4'-(propane-2,2-diyl)diphenol) and 4-tert-octylphenol (OP; 4-(1,1,3,3-tetramethylbutyl)phenol) are among the most common environmental contaminants possessing endocrine disruption properties and are present in plastics, epoxy resins, detergents and other commercial products of common personal and industrial use. A metabolite of BPA, 4-Methyl-2,4-bis(4-hydroxyphenyl)pent-1-ene (MBP) is about 1000 times more biologically active compared to BPA. Epidemiological, clinical, and experimental studies have shown association of BPA and OP with adverse effects on male and female reproductive system in human and animals. The endocrine disruption activity can occur through multiple pathways including binding to steroid receptors. Androgen receptor (AR) and progesterone receptor (PR) are critical for reproductive tract growth and function. Structural binding characterization of BPA, MBP, and OP with AR and PR using molecular docking simulation approaches revealed novel interactions of BPA with PR, and MBP and OP with AR and PR. For BPA, MBP, and OP, five AR interacting residues Leu-701, Leu-704, Asn-705, Met-742, and Phe-764 overlapped with those of native AR ligand testosterone, and four PR interacting residues Leu-715, Leu-718, Met-756, and Met-759 overlapped with those of PR co-complex ligand, norethindrone. For both the receptors the binding strength of MBP was maximum among the three compounds. Thus, these compounds have the potential to block or interfere in the binding of the endogenous native AR and PR ligands and, hence, resulting in dysfunction. The knowledge of the key interactions and the important amino-acid residues also allows better prediction of potential of xenobiotic molecules for disrupting AR- and PR-mediated pathways, thus, helping in design of less potent alternatives for commercial use.

## Introduction

Infertility, defined as a condition when couples are unable to have children, is one of the major problems affecting human health and socio-cultural life. Nearly 72 million couples constituting about 15% of the reproductive-age couples across the world are affected by infertility [1]. Infertility is a public problem and it not only affects the couple’s life but it also affects the health care services and social environment. Depression, grief, guilt, shame, and inadequacy with social isolation are commonly experienced by the infertile couples [2,3]. In general, about 80–85% of cases of infertility are either due to individual male or female factors or due to combination of problems in both partners [4]. The remaining 15–20% cases are due to unexplained infertility and no diagnosis can be made after a complete investigation.

**Fig 1:**
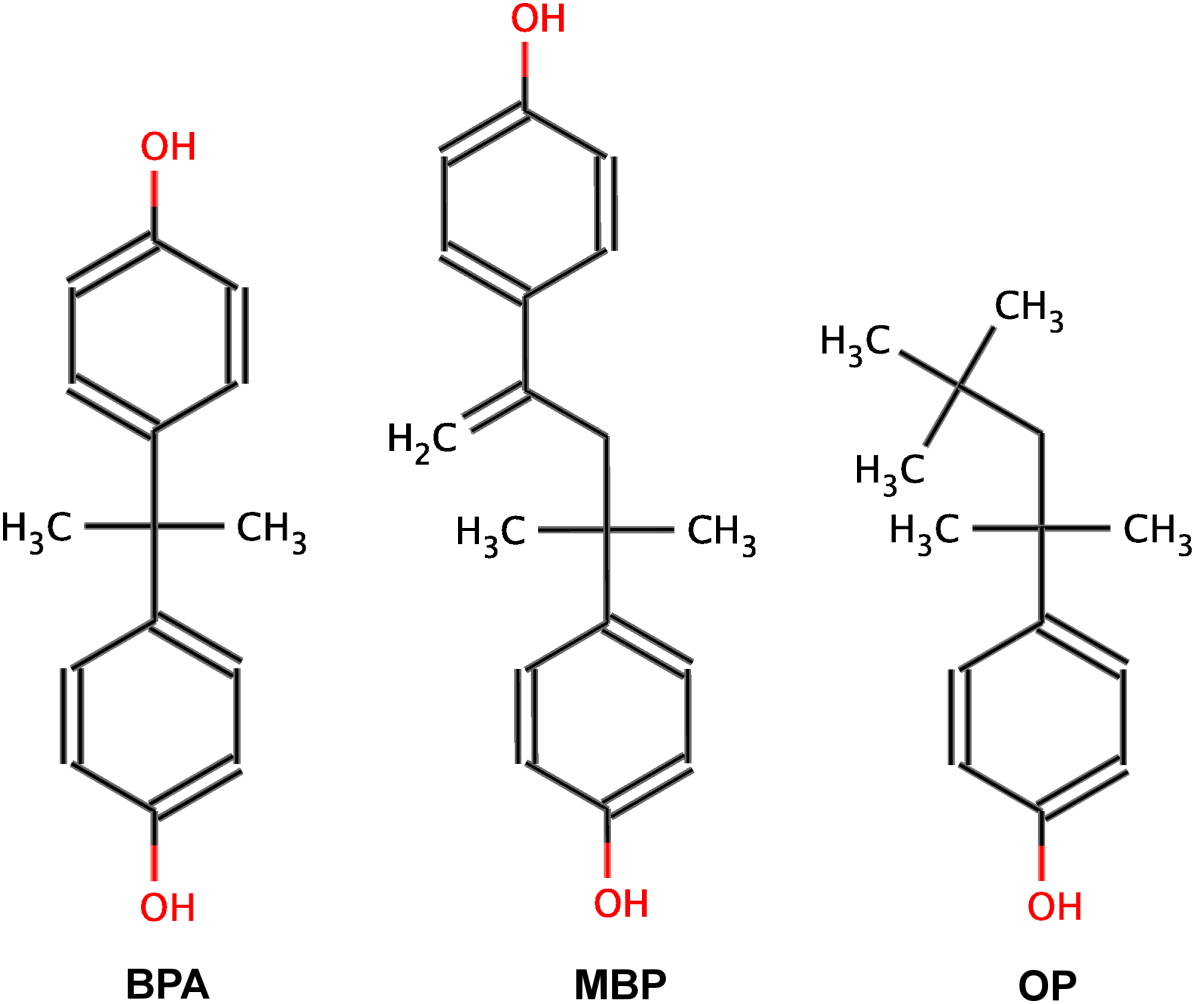
Two dimensional structures of bisphenol A (BPA), 4-Methyl-2,4-bis-(p-hydroxyphenyl)pent-1-ene (MBP), and 4-tert-octylphenol (OP). The hydroxyl groups (-OH) are shown in red color.

Exposure to toxic industrial chemicals that have capacity to disrupt the functions of the endocrine system, also known as endocrine disrupting chemicals (EDCs), in the environment has been increasingly associated with reproductive problems leading to infertility in human population [5,6]. Detection of EDCs in human serum and other fluids has led to the suggestions that these compounds may have adverse effects on the hormonal milieu of the human body leading to various endocrinological and reproductive impairments [7,8]. Bisphenol A (BPA; 4,4'-(propane-2,2-diyl)diphenol) and 4-tert-octylphenol (OP; 4-(1,1,3,3-tetramethylbutyl)phenol) are among the most common environmental contaminants possessing endocrine disruption properties and are known to have weak estrogenic activity. A potent metabolite of BPA, 4-Methyl-2,4-bis(4-hydroxyphenyl)pent-1-ene (MBP) has 1000 times stronger binding activity compared to BPA for estrogen receptors [9,10]. Two-dimensional chemical structures of BPA, MBP, and OP are presented in Fig 1. A study [11] conducted by the Centers for Disease Control and Prevention, USA revealed that 93% of the 2517 human urine samples contained detectable levels of BPA indicating ubiquitous exposure of the human population. BPA is a high production volume chemical used worldwide in the plastic industry and OP is widely used in surfactants, detergents, and cleaners in domestic and industrial products. The annual production of BPA in the world currently is about 8 billion pounds with about 100 tons getting released into the atmosphere each year [12]. The plastics and epoxy resins containing BPA are used in products such as water bottles, baby bottles, eyeglass lenses, medical equipment, toys, CDs/DVDs, cell phones, consumer electronics, household appliances, sports safety equipment, airplanes, and automobiles that impact our daily lives. Epoxy resins containing BPA are used as liners for most food and beverage cans, adhesives, industrial protective coatings, and automotive primers. Many extensive studies have been published on BPA human exposure assessment and epidemiology (reviewed in [13]). The general population is exposed to the BPA through ingestion of contaminated food and drink stored in BPA containing plastic containers, inhalation of contaminated air and dust, and skin contact. A recent report [14] has shown that most human neonates are also exposed to BPA through colostrum and dairy product intake.

Epidemiological and clinical studies have revealed association of BPA with adverse effects on many human systems including the reproductive system [15–17]. Higher BPA levels in women have been associated with poor ovarian response to IVF procedures [18,19], implantation failure [20], infertility [21], polycystic ovary syndrome [22], systemic hormonal imbalance [23,24], endometrial disorders [25], and miscarriage and premature delivery [26,27]. In men, higher BPA levels have been associated with sexual dysfunction [28], lower sperm quality measures [29,30], and hormonal imbalance [31–34]. Genital abnormalities and lower birth weight in male babies [35,36], premature puberty in female children [37], and hormonal imbalance in both male and female children [38] were also associated with higher BPA levels or exposure to BPA contaminated environment. Toxicological studies on mice, rats, and other animal models have also shown that BPA has detrimental effects on the male and female reproductive function (reviewed in [6]). *In vitro* experiments using rat and human fetal testes tissue cultures also showed dose-dependent anti-androgenic effects of BPA [39].

*In vivo* studies on OP in the laboratory animals have also demonstrated adverse effects such as altered sex hormone levels and hypothalamic-pituitary suppression, impaired steroidogenesis, altered estrous cycles and reproductive outcomes, altered neonatal sexual development, testicular atrophy, and impaired spermatogenesis [40–43]. *In vivo* studies with BPA and OP in immature mice have shown potential progestogenic activity on uterus [44].

The endocrine disruption activity of many of the compounds including BPA has been proposed to be due to agonistic and antagonistic interference with the steroidal pathways involved in the development and function of male and female reproductive system [45]. These interferences can occur through multiple pathways including binding to steroid receptors and steroid binding proteins or modulating steroidogenic enzymes in the body. Binding of the EDCs such as BPA, BPA metabolite MBP, or OP to androgen receptors (AR) and progesterone receptors (PR) in the body represents a potential way of interfering in the natural ligand-protein interactions and thus leading to harmful ramification for the normal functioning of the steroid target organs. The AR and PR are closely related members of the nuclear receptor superfamily. The principle steroid ligands for AR are testosterone and dihydrotestosterone and AR signaling is important for developmental and physiological processes especially male sexual development and maturation as well as maintenance of male reproductive organs and spermatogenesis [46]. The PR through activation by the progesterone plays a central role in diverse reproductive events associated with establishment and maintenance of pregnancy, ovarian function, alveolar development in the breast, and sexual behavior [47]. *In vitro* screening using hormone responsive reporter assays and binding studies have identified BPA as potent AR ligand with anti-androgenic activity [48–51]. *In silico* molecular modelling of human AR with BPA has also been performed [51–54]. However, molecular interactions of MBP and OP with AR and BPA, MBP, and OP with PR have not been characterized.

The current study is an attempt at the structural binding characterization of BPA, MBP, and OP with AR and PR using molecular docking simulation approaches. The details of binding mechanism of three disruptors were delineated individually and then comparisons of the distinctive binding pattern and their interacting residues was done. In view of the reported adverse effects of the three EDCs on human health and reproduction, the current study is an important step further for gaining an insight into their potential interfering mechanisms of reproductive processes.

## Materials and Methods

### Data retrieval

The molecular structures of BPA, MBP, and OP were retrieved from PubChem compound database with Compound IDs CIDs 6623, 83494, and 8814, respectively. The X-ray crystal structure of ligand binding domains of human AR (PDB Id: 2AM9) with bound testosterone (TST) and human PR (PDB Id: 1SQN) with bound norethindrone (NET) were obtained from Protein Data Bank (PDB, http://www.rcsb.org/). Both the PDB structures were co-complexes with the respective bound ligands that acted as clues for identifying catalytic sites which were used in molecular docking.

### Molecular docking

The molecular docking simulations of BPA, MBP, and OP into the binding sites of AR and PR were carried out by Dock v.6.5 (University of California, San Francisco, USA) [55]. The strategy for identifying the best docked pose involved Random Conformation Search which utilizes the grid-based scoring functions of Coulombic and Lennard-Jones forces. The initial structure preparation of proteins and ligands required for docking and visual analyses at different stages were performed by Chimera v.1.6.2 [56].

### Analyses of docked receptor-ligand complex

For visual analyses and illustrations of the receptor-ligand complexes, PyMOL v.1.3 was used [57]. The polar and hydrophobic interactions between the receptors and the ligands were analyzed and the illustrations were generated by Ligplot+ v.1.4.3 program [58,59]. To score the extent of involvement of residues in binding, loss in Accessible Surface Area (ASA) due to ligand binding was evaluated. For a residue to be involved in binding it should lose more than 10 Å^2^ ASA in the direction from unbound to the bound state [60]. The ASA calculations of unbound receptor and the receptor-ligand complex were performed by Naccess v.2.1.1 [61]. The following expression yields the loss in ASA (ΔASA) of the *i* ^th^ residue due to ligand binding:

$$\Delta ASA_{i}\;=\;ASA_{i}^{Unbound\;receptor}-\;ASA_{i}^{Receptor-ligand\;complex}$$

where ASA_i_^Unbound receptor^ is the ASA of the *i* ^th^ residue in unbound receptor i.e., the receptor without ligand and ASA_i_^Receptor-ligand complex^ is the ASA of the *i* ^th^ residue in bound receptor i.e., the receptor with bound ligand.

In addition to the Dock score (Grid score) obtained from Dock v.6.5 [55], the calculation of the binding energies and dissociation constants were also carried out by X-Score v.1.2.11 [62,63].

### Protein sequence alignment and analyses

The amino acid sequences of ligand binding domains of AR and PR were aligned using Muscle v.3.8.31 [64], and further analyses and illustration were performed by Jalview v.2.8 [65,66].

## Results and Discussion

### Molecular docking studies of BPA, MBP, and OP with AR

**Fig 2:**
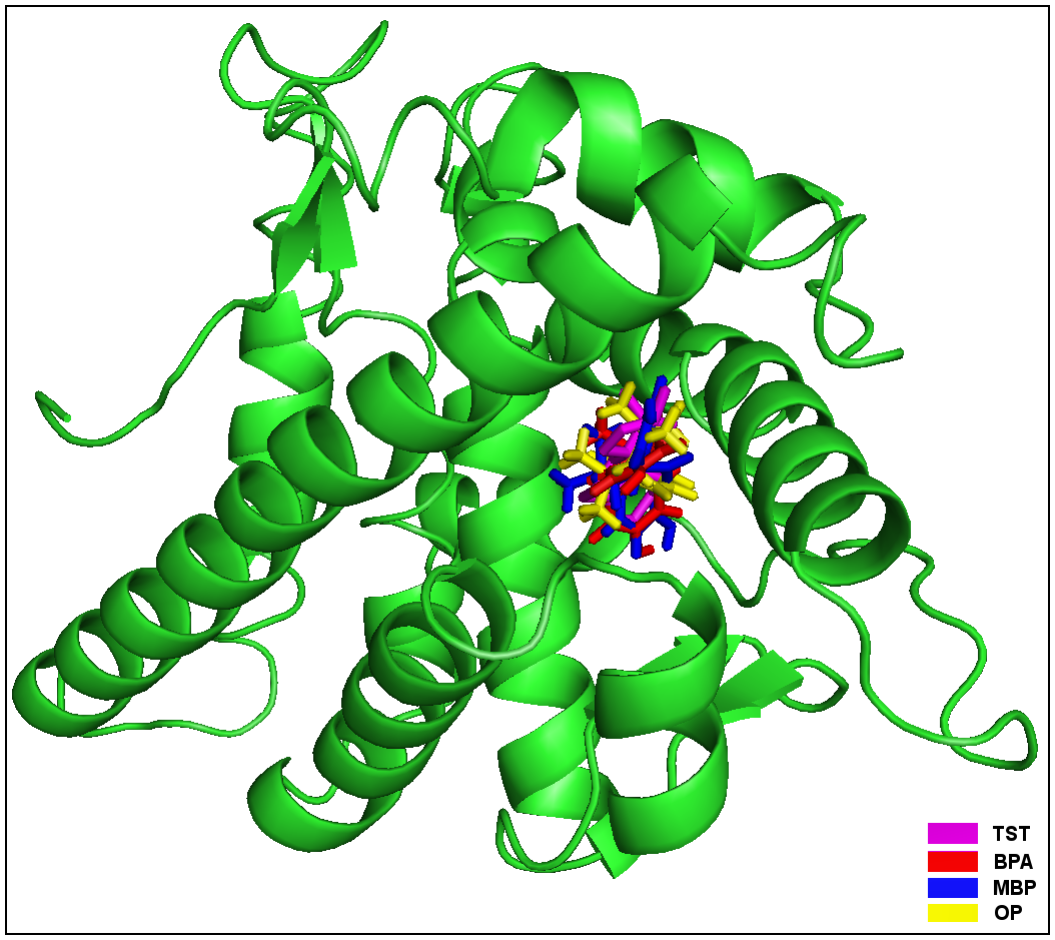
Human androgen receptor (AR) is illustrated in cartoon representation and bisphenol A (BPA), methylbishydroxyphenylpentene (MBP), 4-tert-octylphenol (OP), and natural ligand testosterone (TST) are in stick representation in different colors.

**Fig 3:**
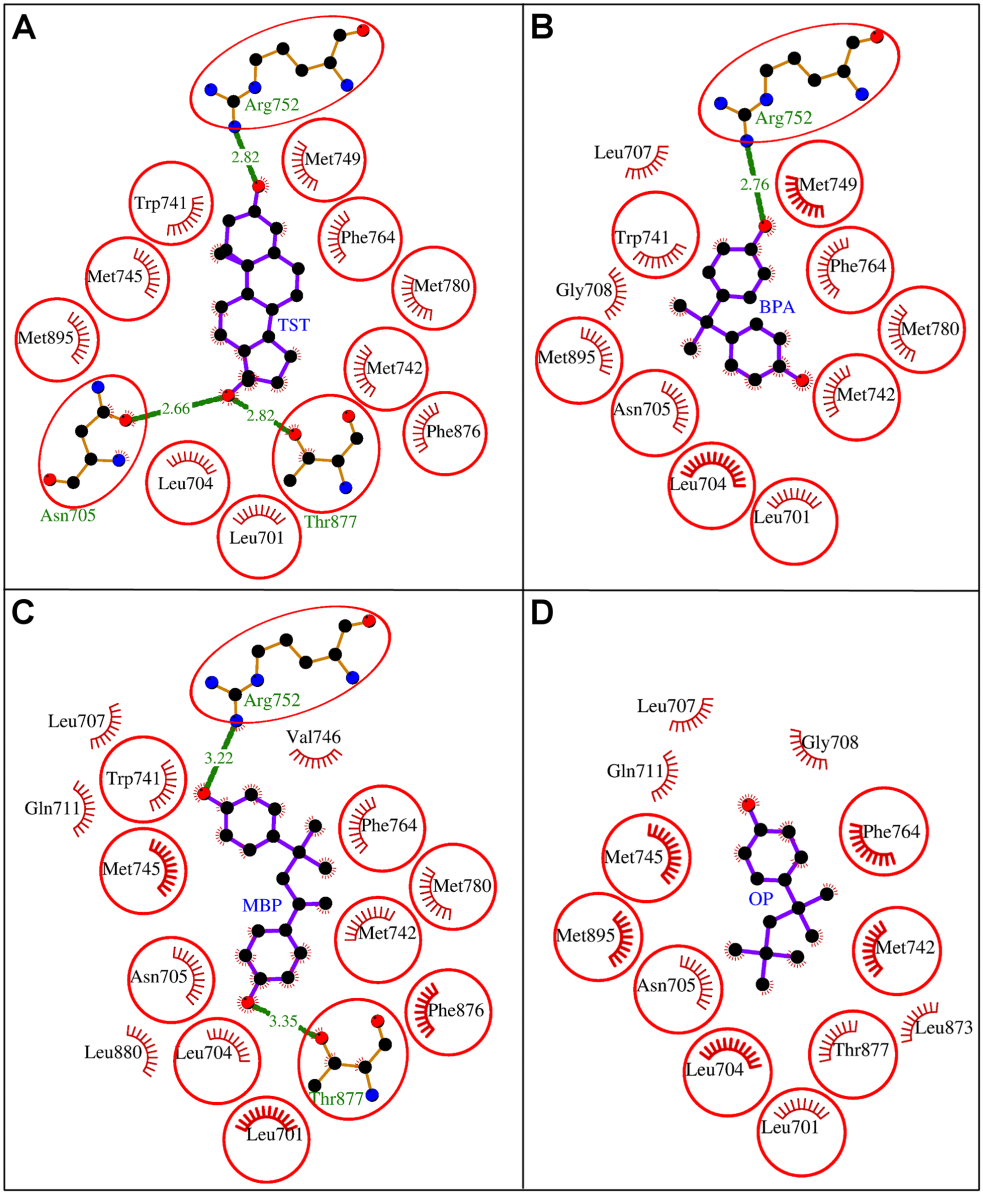
Comparative binding analysis of A: natural ligand testosterone (TST) with B: bisphenol A (BPA), C: methylbishydroxyphenylpentene (MBP), and D: 4-tert-octylphenol (OP) in the binding site of human androgen receptor (AR). The hydrogen bonds are shown as green-dashed lines with indicated bond lengths and the residues involved in hydrophobic interactions are shown as red arcs. The interacting residues which are common for all the ligands are encircled.

The molecular docking study revealed that the BPA packed against AR residues Leu-701, Leu-704, Asn-705, Leu-707, Gly-708, Trp-741, Met-742, Met-749, Arg-752, Phe-764, Met-780, and Met-895. These 12 residues stabilized the BPA-AR complex by exerting 12 hydrophobic interactions and single hydrogen bond (Figs 2 and 3, Table 1). Further, the high values of Dock score, binding energy, and dissociation constant assured good quality docking, however, the binding energy and dissociation constant were less compared to those of the bound natural ligand, TST (Table 2). The residue Leu-704 was identified as the key interacting residue as it showed maximum number of hydrophobic contacts with BPA and maximum ΔASA (loss in Accessible Surface Area) due to BPA binding (Table 1). The residue Arg-752 formed an H-bond (2.76 Å) with phenolic oxygen atom of BPA through guanidinium N-atom (Fig 3B). When the binding of the docked BPA to AR was compared with that of the bound TST, all the BPA interacting residues were overlapping except Leu-707 and Gly-708 (Fig 3B). The consistent involvement of BPA interacting residues with those of TST provide weight to accuracy of our docking simulations. This also showed that BPA too interacted with the important residues of AR common to those of the bound TST and, thus, has potential for interference in the receptor function.

**Table 1:** The human androgen receptor (AR) residues interacting with bisphenol A (BPA) are listed with the number of hydrophobic interactions and loss in Accessible Surface Area (ΔASA). The ranking of residues on the basis of ΔASA is indicated by superscripts with the value of ΔASA.

Interacting residues	No. of hydrophobic interactions	ΔASA (Å^2^)
Leu-701	1	6.74^8^
Leu-704	6	24.02^1^
Asn-705	1	6.73^9^
Leu-707	2	10.08^5^
Gly-708	2	8.11^7^
Trp-741	1	5.44^10^
Met-742	1	18.4^2^
Met-749	2	4.48^12^
Arg-752	H-bonding	5.28^11^
Phe-764	5	17.65^3^
Met-780	4	10.96^4^
Met-895	1	9.39^6^

**Table 2:** The binding strengths of bisphenol A (BPA), BPA metabolite methylbishydroxyphenylpentene (MBP), and 4-tert-octylphenol (OP) with human androgen receptor (AR) and progesterone receptor (PR) are shown with the number of molecular interactions and other scores. Testosterone (TST) and norethindrone (NET), respectively, are the bound ligands co-complexed with the PDB structures of the two indicated receptors. The number of residues involved in the hydrophobic interactions are provided in parentheses. The 'K_d_' denotes the dissociation constant. The binding energy and pK_d_ or −log(K_d_) values are calculated using X-Score. The more negative is the Dock/Grid score, the better is the docking.

Target	PDB ID	Ligand	Hydrogen bonds	Hydrophobic interactions	Dock/Grid score	Binding energy (kcal/Mol)	pK_d_
AR	2AM9	BPA	1	26 (12)	-20.21	-8.77	6.43
		MBP	-	37 (15)	-18.92	-9.46	6.93
		OP	-	25 (12)	-21.61	-8.76	6.42
		TST	3	26 (13)	-42.93	-10.34	7.58
PR	1SQN	BPA	-	18 (9)	-33.37	-8.74	6.41
		MBP	1	24 (12)	-42.27	-9.38	6.87
		OP	-	17 (11)	-29.71	-8.50	6.23
		NET	1	29 (14)	-44.44	-9.70	7.11

In the molecular docking study of MBP with AR, the MBP packed against the residues Leu-701, Leu-704, Asn-705, Leu-707, Gln-711, Trp-741, Met-742, Met-745, Val-746, Arg-752, Phe-764, Met-780, Phe-876, Thr-877, and Leu-880 in the binding site (Figs 2 and 3, Table 3). Altogether these 15 residues exerted 37 hydrophobic and two hydrogen bonding interactions with MBP and stabilized the MBP-AR complex (Fig 3C, Table 3). The high values of Dock score, binding energy, and dissociation constant assured good quality docking, however, the binding energy and the dissociation constant were less than that of the natural ligand, TST (Table 2). The residue Leu-704 of AR was identified as the key interacting residue as it showed maximum ΔASA due to MBP binding and exerted maximum number of hydrophobic contacts (Table 3). The residue Arg-752 through guanidinium N-atom formed an H-bond (3.22 Å) with one phenolic oxygen atom of MBP, whereas, Thr-877 through hydroxyl oxygen of its side chain formed another H-bond (3.35 Å) with another phenolic oxygen of MBP (Fig 3C). The comparison of the AR binding of the docked MBP with that of the bound TST revealed that 11 of 15 residues were overlapping among the interacting residues of both the ligands (Fig 3C). Further, the two hydrogen bonds formed with MBP were also common with those of TST. This provided support to our docking simulation accuracy and also suggested that MBP was also blocking important TST interacting residues and, thus, has potential for interference in AR function.

**Table 3:** The human androgen receptor (AR) residues interacting with bisphenol A metabolite methylbishydroxyphenylpentene (MBP) are listed with the number of hydrophobic interactions and loss in Accessible Surface Area (ΔASA). The ranking of residues on the basis of ΔASA is indicated by superscripts with the value of ΔASA.

Interacting residues	No. of hydrophobic interactions	ΔASA (Å^2^)
Leu-701	3	6.99^10^
Leu-704	4	24.02^1^
Asn-705	3	7.31^8^
Leu-707	4	10.08^7^
Gln-711	3	5.1^13^
Trp-741	1	5.64^12^
Met-742	2	18.72^3^
Met-745	3	22.05^2^
Val-746	1	7.1^9^
Arg-752	1	5.77^11^
Phe-764	3	17.65^4^
Met-780	3	10.96^6^
Phe-876	1	3.73^14^
Thr-877	4	14.23^5^
Leu-880	1	1.41^15^

The molecular docking of OP with AR showed that OP was sitting in the binding site of AR and packing it against the residues Leu-701, Leu-704, Asn-705, Leu-707, Gly-708, Gln-711, Met-742, Met-745, Phe-764, Leu-873, Thr-877, and Met-895 (Figs 2 and 3, Table 4). These 12 interacting residues together exerted 25 hydrophobic interactions and stabilized the OP-AR complex. The high values of the Dock score, the binding energy, and dissociation constant showed good quality binding of OP with AR, however, as with BPA and MBP the binding energy and dissociation constant were less compared to those of the natural ligand, TST (Table 2). The residue Leu-704 of AR was identified as the key residue in OP binding as it showed maximum ΔASA due to binding and formed maximum number of hydrophobic interactions (Table 4). On comparing the binding of OP to AR with that of the bound TST, eight residues (Leu-701, Leu-704, Asn-705, Met-742, Met-745, Phe-764, Thr-877, and Met-895) were found overlapping among the interacting residues of both the ligands (Fig 3D). This showed that OP interacted with the residues important for TST binding and, thus, indicating a potential for interference in the function of AR.

**Table 4:** The human androgen receptor (AR) residues interacting with 4-tert-octylphenol (OP) are listed with the number of hydrophobic interactions and loss in Accessible Surface Area (ΔASA). The ranking of residues on the basis of ΔASA is indicated by superscripts with the value of ΔASA.

Interacting residues	No. of hydrophobic interactions	ΔASA (Å^2^)
Leu-701	1	6.99^11^
Leu-704	5	24.02^1^
Asn-705	2	7.31^10^
Leu-707	4	9.76^8^
Gly-708	1	8.11^9^
Gln-711	2	3.81^12^
Met-742	2	19.55^2^
Met-745	3	17.92^3^
Phe-764	1	15.63^4^
Leu-873	1	10.44^6^
Thr-877	2	14.66^5^
Met-895	1	10.06^7^

Summarizing the docking results of the three compounds, BPA, MBP, and OP, the five residues Leu-701, Leu-704, Asn-705, Met-742, and Phe-764 were overlapping with those of TST. The Leu-704 was consistently identified as the key interacting residue for all the three compounds. The binding strengths of the three compounds for AR were less compared to that of TST. However, the binding strength of MBP for AR was maximum among the three compounds, while those of BPA and OP were comparable based on Dock/grid score, binding energy, and dissociation constants (Table 3).

The BPA docking simulations with AR in this study support and confirm the *in silico* findings of previous molecular modelling studies of human AR with BPA [51–54]. In one study [54], docking and CoMSIA was performed on 45 non-steroidal compounds including BPA as AR ligands and the reported important interacting residues Leu-701, Leu-704, Asn-705, Met-742, and Arg-752 were overlapping with the interacting residues for BPA in the current study. However, MBP and OP were not among the selected compounds. *In silico* modelling of AR with MBP and OP has not been reported previously. However, the overlapping interacting residues for the BPA in this and the previous study [54] were also similar to the interacting residues for MBP and OP in this study, as noted above. The potential BPA-AR interactions as indicated by docking simulations have also been confirmed previously by *in vitro* methods. Using luciferase reporter assays BPA was shown as a potent AR ligand with anti-androgenic activity [49–51]. Similar *in vitro* ligand binding studies of AR with MBP and OP have not been reported. However, *in silico* modelling and *in vitro* luciferase reporter assays have shown that MBP was a more potent binder of estrogen receptor (another steroid receptor) than BPA [9,10,67].

### Molecular docking studies of BPA, MBP, and OP with PR

**Fig 4:**
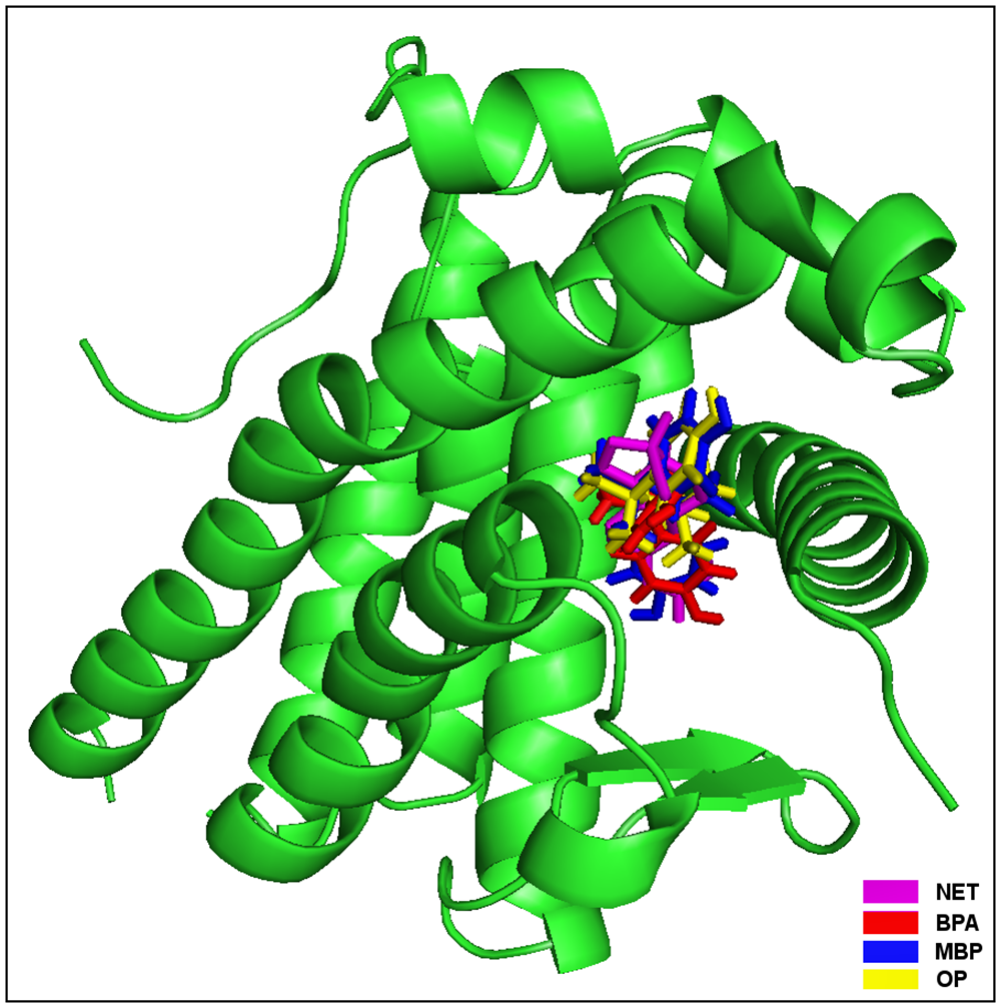
Human progesterone receptor (PR) is illustrated in cartoon representation and bisphenol A (BPA), methylbishydroxyphenylpentene (MBP), 4-tert-octylphenol (OP), and the bound ligand norethindrone (NET) are in stick representation in different colors.

**Fig 5:**
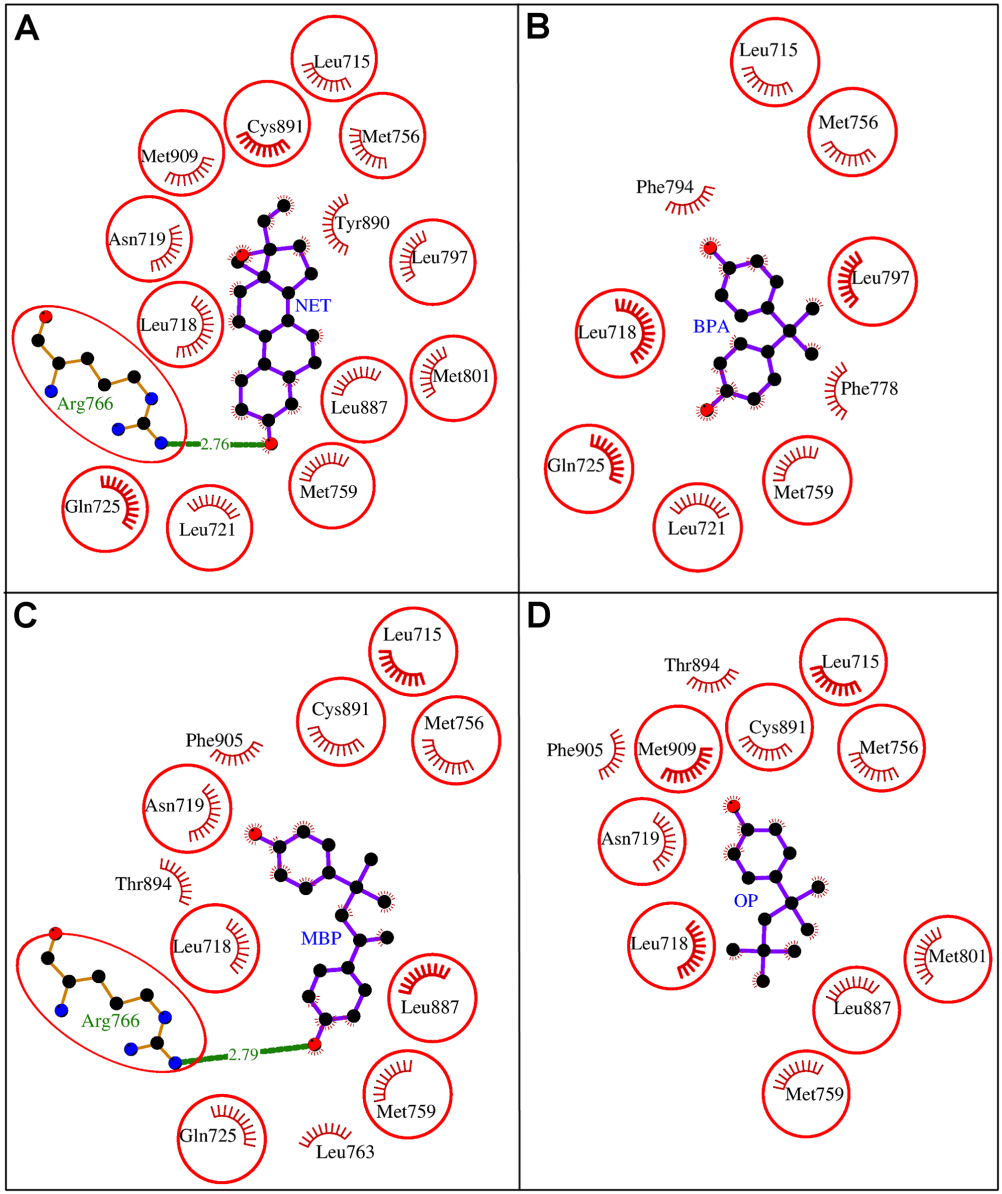
Comparative binding analysis of A: bound ligand norethindrone (NET) with B: bisphenol A (BPA), C: methylbishydroxyphenylpentene (MBP), and D: 4-tert-octylphenol (OP) in the binding site of human progesterone receptor (PR). The hydrogen bonds are shown as green-dashed lines with indicated bond lengths and the residues involved in hydrophobic interactions are shown as red arcs. The interacting residues which are common for all the ligands are encircled.

The molecular docking study of BPA with PR identified the following BPA interacting residues of PR: Leu-715, Leu-718, Leu-721, Gln-725, Met-756, Met-759, Phe-778, Phe-794, and Leu-797 (Figs 4 and 5, Table 5). The indicated nine residues together formed 18 hydrophobic interactions and stabilized the BPA-PR complex. Further, the high values of the Dock score, binding energy, and dissociation constant also showed good quality of binding, however, binding energy, and dissociation constant were less compared to those of the bound ligand NET from PDB PR co-complex structure (Table 2). The residue Leu-718 was identified as the key interacting residue as it showed maximum number of hydrophobic contacts and maximum ΔASA due to BPA binding (Table 5). The comparison in binding of BPA to PR with the bound NET showed that all the BPA interacting residues were overlapping except Phe-778 and Phe-794 (Fig 5B). The overlapping of residues suggested that BPA has potential to occupy important residues in different molecular interactions and hence not making them available for binding of natural ligand to PR. Thus, BPA has potential for interference in the normal function of PR.

**Table 5:** The human progesterone receptor (PR) residues interacting with bisphenol A (BPA) are listed with the number of hydrophobic interactions and loss in Accessible Surface Area (ΔASA). The ranking of residues on the basis of ΔASA is indicated by superscripts with the value of ΔASA.

Interacting residues	No. of hydrophobic interactions	ΔASA (Å^2^)
Leu-715	1	7.66^6^
Leu-718	4	29.6^1^
Leu-721	3	5.57^7^
Gln-725	2	5.44^8^
Met-756	2	18.85^3^
Met-759	1	21.29^2^
Phe-778	3	17.45^4^
Phe-794	1	1.29^9^
Leu-797	1	12.68^5^

The MBP interacting residues of PR identified in the current study were Leu-715, Leu-718, Asn-719, Gln-725, Met-756, Met-759, Leu-763, Arg-766, Leu-887, Cys-891, Thr-894, and Phe-905 (Figs 4 and 5, Table 6). Altogether, the 12 interacting residues formed 24 hydrophobic and single hydrogen bonding interactions with MBP and, thus, stabilized the MBP-PR complex. The high values of Dock score, the binding energy, and dissociation constant showed good quality docking and the values were also comparable to that of the bound ligand, NET (Table 2). The residue Leu-718 showed maximum ΔASA due to MBP binding, whereas, the residues Asn-719, Gln-725, and Met-759 were involved in maximum number of hydrophobic contacts with MBP. Another important residue Arg-766 was involved in a hydrogen-bonding interaction (2.79 Å) through guanidinium N-atom with one phenolic oxygen atom of MBP (Fig 5C). The comparison of binding of MBP to PR with the bound NET showed that 9 of 12 residues were overlapping among the interacting residues of both the ligands. Further, the only hydrogen bond formed with MBP was also common with that of NET. This suggested that MBP was also occupying the important NET interacting residues and, thus, indicating its potential for interfering with PR function.

**Table 6:** The human progesterone receptor (PR) residues interacting with bisphenol A metabolite methylbishydroxyphenylpentene (MBP) are listed with the number of hydrophobic interactions and loss in Accessible Surface Area (ΔASA). The ranking of residues on the basis of ΔASA is indicated by superscripts with the value of ΔASA.

Interacting residues	No. of hydrophobic interactions	ΔASA (Å^2^)
Leu-715	1	9.96^6^
Leu-718	1	29.6^1^
Asn-719	4	12.81^5^
Gln-725	4	5.44^8^
Met-756	2	19.52^3^
Met-759	4	21.29^2^
Leu-763	1	4.87^10^
Arg-766*	H-bonding	2.11^12^
Leu-887	1	8.06^7^
Cys-891	3	13.31^4^
Thr-894	2	5.09^9^
Phe-905	1	2.3^11^

The molecular docking study of OP with PR showed that OP was sitting in the binding site using interacting residues Leu-715, Leu-718, Asn-719, Met-756, Met-759, Met-801, Leu-887, Cys-891, Thr-894, Phe-905, and Met-909 (Figs 4 and 5, Table 7). These 11 interacting residues together exerted 17 hydrophobic interactions and stabilized the OP-PR complex. The values of the dock score, the binding energy, and dissociation constant showed good quality binding of OP with PR, however, the binding energy and dissociation constant were less compared to those of the bound ligand, NET (Table 2). The Leu-718 was identified as the key residue in OP binding to PR as it showed maximum ΔASA due to binding (Table 7). The comparison of OP binding to PR with that of bound NET revealed nine overlapping residues among the interacting residues of both the ligands (Fig 5). This suggested that OP has the potential to interfere with binding of important residues for NET binding and thereby, indicating its potential for interference with the normal function of PR.

**Table 7:** The human progesterone receptor (PR) residues interacting with 4-tert-octylphenol (OP) are listed with the number of hydrophobic interactions and loss in Accessible Surface Area (ΔASA). The ranking of residues on the basis of ΔASA is indicated by superscripts with the value of ΔASA.

Interacting residues	No. of hydrophobic interactions	ΔASA (Å^2^)
Leu-715	1	9.96^8^
Leu-718	2	29.6^1^
Asn-719	2	12.81^5^
Met-756	1	19.24^2^
Met-759	2	17.64^3^
Met-801	2	11.82^7^
Leu-887	1	8.00^9^
Cys-891	2	13.31^4^
Thr-894	2	5.09^10^
Phe-905	1	2.3^11^
Met-909	1	12.48^6^

Summarizing the docking of the three compounds, BPA, MBP, and OP to PR, the four residues Leu-715, Leu-718, Met-756, and Met-759 were overlapping with those of bound ligand, NET. The binding strength of MBP was comparable to that of the bound NET. However, among the three compounds the binding strength of MBP was maximum, followed in order by BPA and OP based on dock/grid score, binding energy, and dissociation constants (Table 7). The information about the binding mode and the interacting residues of PR with BPA, MBP, and OP is novel and studies regarding this have not been reported previously. *In vitro* ligand binding studies of PR with BPA, MBP, and OP have also not been reported.

### Comparative analyses of binding sites of AR and PR

**Fig 6:**
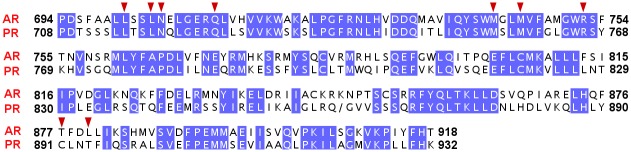
Sequence alignment of ligand binding sites of human androgen receptor (AR) and progesterone receptor (PR). The amino acids showing sequence identity in both the receptors are shown as white text with blue background, whereas, the rest of the amino acids are shown in black text. The initial and final position of each receptor in the alignment is also provided. The position-equivalent-residues (residues of both receptors falling at similar column position in the alignment) overlapping among the interacting residues of methylbishydroxyphenylpentene (MBP), are marked by red triangles.

**Fig 7:**
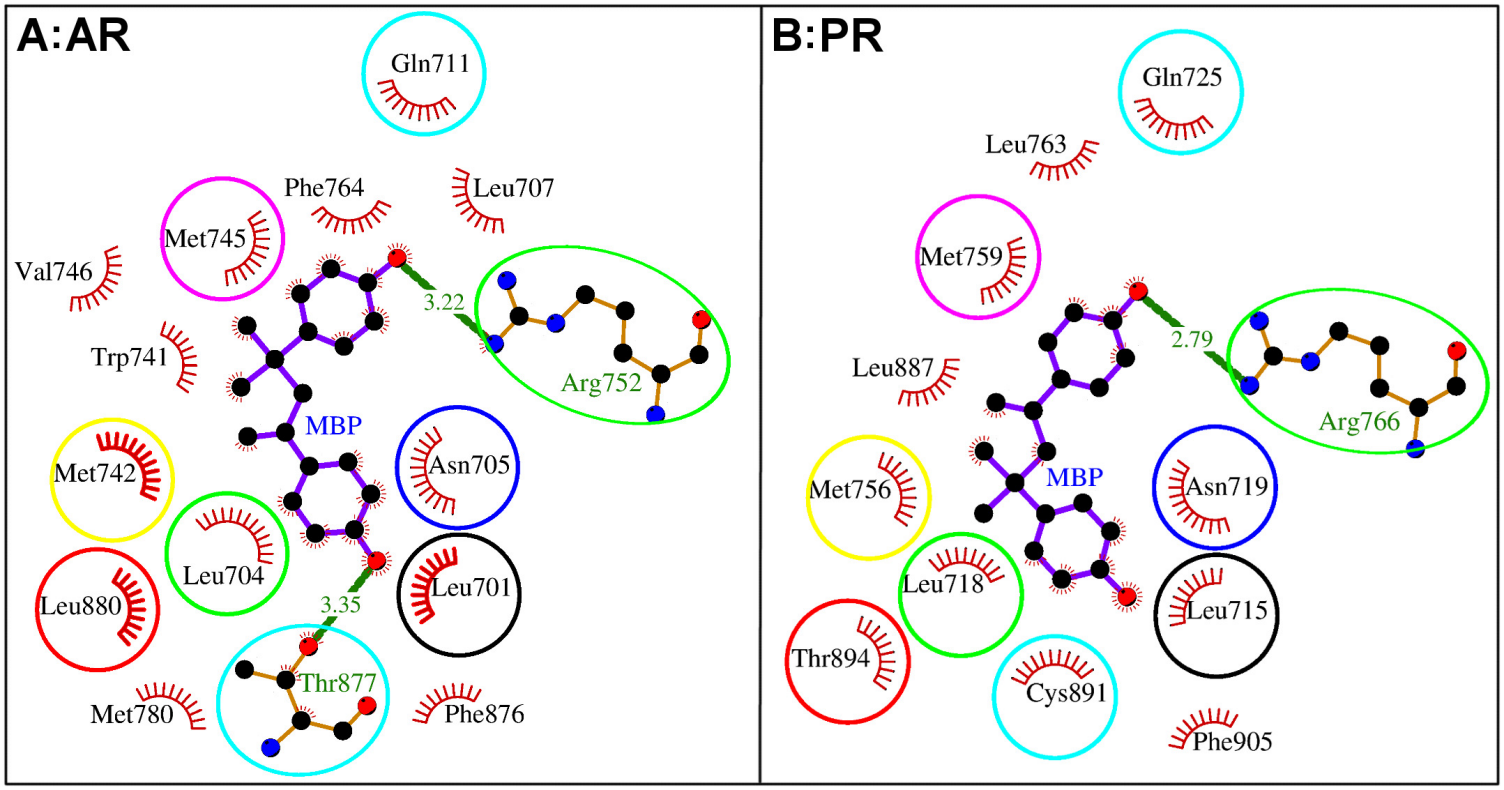
Comparative analysis of methylbishydroxyphenylpentene (MBP) binding to human androgen receptor (AR) and progesterone receptor (PR). Panel A and Panel B display the interacting residues of AR and PR, respectively, with MBP when MBP is kept in the same orientation. On visual analyses in PyMol, the interacting residues were superimposed keeping the docked MBP in same orientation. The residues of the two receptors which were falling at similar location with respect to MBP were encircled in similar color.

In order to compare the binding sites of AR and PR, the binding domains of the two steroid receptors were aligned and the equivalent residues (residues of both receptors falling at similar column position in the alignment) were identified between the two receptors (Fig 6). From the docking studies, MBP was identified as the best binder among the three chosen compounds, BPA, MBP, and OP for both AR and PR. For comparison within the binding site, the docked MBP was chosen and kept in the same orientation and the interacting residues of AR and PR were superimposed (Fig 7). The residues falling at the similar location with respect to the docked MBP, were again cross-checked in the list of equivalent residues from domain alignment. The results from both the alignment and the structure were corroborating with each other and we obtained the same list of position-equivalent residue pairs (Table 8). The MBP interacting residues from both the receptors were compared resulting in identification of nine position-equivalent residue pairs of which seven pairs were identical (Figs 6 and 7, Table 8). It is worth noting that the hydrogen bonding by position equivalent Arg (Arg-752 in AR and Arg-766 in PR) were also common. Further, the position-equivalent residue Leu (Leu-704 in AR and Leu-718 in PR) have shown maximum ΔASA underscoring their importance in binding. The high sequence identity among the residues in the binding site explains the binding of compounds with common steroid scaffold.

**Table 8:** The equivalent residue pairs of bisphenol metabolite methylbishydroxyphenylpentene (MBP) interacting residues from human androgen receptor (AR) and progesterone receptors (PR) are listed. All the listed equivalent residue pairs showed sequence identity except the two pairs shown in bold.

AR	PR
Leu-701	Leu-715
Leu-704^#^	Leu-718^#^
Asn-705	Asn-719
Gln-711	Gln-725
Met-742	Met-756
Met-745	Met-759
Arg-752*	Arg-766*
**Leu-880**	**Thr-894**
**Thr-877**	**Cys-891**

^#^ The residues which showed maximum loss in Accessible Surface Area (ΔASA)

* The residues which form hydrogen bond with MBP.

## Conclusions

The present study used docking simulation analyses to identify interacting residues of human AR and PR and to delineate the details of binding mechanism of three endocrine disruptors BPA, MBP, and OP individually as well as comparing their distinctive binding pattern. The study showed novel interactions of BPA with PR, and MBP and OP with AR and PR. For BPA, MBP, and OP, five AR interacting residues Leu-701, Leu-704, Asn-705, Met-742, and Phe-764 overlapped with those of native AR ligand TST, and four PR interacting residues Leu-715, Leu-718, Met-756, and Met-759 overlapped with those of PR co-complex ligand, NET. For both the receptors the binding strength of MBP was maximum among the three compounds. Superimposition of interacting residues of AR and PR for the docked MBP in the binding site resulted in identification of nine position-equivalent residue pairs of which seven pairs were identical. The high sequence identity among the residues of AR and PR in the binding site explains the binding of compounds with common steroid scaffold. Thus, these compounds have the potential to block or interfere in the binding of the endogenous native AR and PR ligands and, hence, resulting in dysfunction of the AR and PR signaling. In view of the reported adverse effects of the three EDCs on male and female reproductive function, the current study is an important step further for gaining an insight into their potential interfering mechanisms in human reproductive processes. Further, the knowledge of key interactions and important amino-acid residues also allows better prediction of potential of xenobiotic molecules for disrupting AR- and PR-mediated pathways, thus, helping in design of safe alternatives for commercial use.
